# Evaluation of Parents and Child Satisfaction Toward Primary Molar Restoration with Preformed Metal Crowns and Its Impact on Child Bullying

**DOI:** 10.3390/healthcare14010062

**Published:** 2025-12-26

**Authors:** Abdulfatah Alazmah

**Affiliations:** Department of Pediatric Dentistry, College of Dentistry, Prince Sattam bin Abdulaziz University, Al-Kharj 11942, Saudi Arabia; a.alazmah@psau.edu.sa

**Keywords:** child bullying, primary teeth, metal crowns, parents

## Abstract

**Objective:** This study aimed to assess the psychosocial impact of stainless steel crowns (SSCs) among primary school children in Dammam, Saudi Arabia, focusing on bullying experiences, child satisfaction, and parental perception. **Methods:** A cross-sectional survey was conducted in June 2025 among 123 children (mean age 7.8 years; 52% male) from two randomly selected schools. Schools were chosen using simple random sampling from a Ministry of Education-approved list. All children aged 6–10 years with at least one SSC placed for six months or more were eligible. A validated, self-administered questionnaire completed by children and their parents assessed bullying related to SSCs, satisfaction with the crown’s appearance, and perceived impact. Statistical analyses included descriptive statistics, Chi-square tests, and logistic regression to evaluate associations between variables (*p* < 0.05). **Results:** Bullying was reported by 39.0% of children, primarily verbal (58.1%), followed by social exclusion (29.1%). Although gender differences in bullying were not statistically significant (*p* = 0.829), boys more often reported nickname-based teasing (*p* < 0.001). Only 35.0% of children were satisfied with the crown’s shape and 29.3% with its color. Nearly half (48.8%) felt uncomfortable when asked about it. In contrast, parental satisfaction was higher (69.1%), though only 42.3% believed their child had fully accepted the crown. **Conclusions:** While SSCs are clinically effective and accepted by most parents, a notable proportion of children experience bullying and aesthetic dissatisfaction. These findings highlight the need for child-centered care and consideration of esthetic alternatives.

## 1. Introduction

Bullying in schools is a global issue that must be stopped due to its serious mental health School bullying is a pervasive global issue with well-documented psychological, emotional, and academic consequences. It can manifest in both direct forms—such as physical aggression (hitting, kicking) and verbal harassment (name-calling, threats)—and indirect forms, including social exclusion, rumor spreading, and manipulation of peer relationships. Verbal bullying, particularly name-calling, is the most prevalent and insidious form due to its repetitive and psychologically damaging nature [1].

Although bullying may occur in various settings, it most commonly takes place in or around schools [2,3]. A cross-national study conducted among 8–18-year-olds across 11 European countries found that approximately 20.6% reported experiencing bullying, with prevalence estimates varying widely depending on age group, geographical region, and study methodology. The Global School-based Student Health Survey (GSHS) also reported a bullying prevalence ranging from 21% to 64% across 19 low- and middle-income countries, highlighting the magnitude of this issue [4,5].

Bullying has been consistently associated with negative mental health outcomes such as anxiety, depression, reduced self-esteem, and poor academic performance. Long-term consequences may also include social withdrawal, behavioral issues, and criminal activity. A child’s mental well-being and personality development are influenced by their social environment, and exposure to bullying can hinder healthy emotional growth [6].

Dental characteristics that deviate from the perceived norm, such as malocclusions—including anterior open bites—have been consistently linked to negative social outcomes in children, including teasing and bullying. These features can affect speech, facial aesthetics, and self-perception, making affected children more vulnerable to peer victimization. Similarly, restorative interventions like stainless steel crowns (SSCs), while functionally effective, may contribute to psychosocial distress due to their conspicuous metallic appearance [7]. The visibility of such restorations can draw unwanted attention, leading to social exclusion or verbal harassment. Together, both naturally occurring dental anomalies and visibly distinguishable restorations highlight the significant role of oral aesthetics in shaping children’s peer interactions and emotional well-being.

Given the influence of dental appearance on children’s psychosocial well-being, the type of restorative treatment selected can play a critical role in both oral health and social adjustment. In pediatric dentistry, SSCs have been a widely accepted restorative solution for severely decayed primary molars for over 70 years. Despite their clinical durability, cost-effectiveness, and ease of placement, SSCs have notable aesthetic limitations due to their metallic appearance [8]. Consequently, preformed zirconia crowns (PZCs) have gained popularity as a more aesthetically pleasing alternative. However, SSCs remain the standard choice for multi-surface lesions and pulpotomized molars globally due to their proven efficacy [9,10,11,12].

A recent study highlights the significant role of dental appearance in shaping children’s social experiences and psychological well-being [13]. In particular, visible dental restorations, such as SSCs, have been associated with teasing, social exclusion, and bullying in school settings, all of which can negatively affect children’s oral-health-related quality of life (OHRQoL) [14,15]. These psychosocial effects, including decreased self-esteem, peer avoidance, and emotional distress, are of growing concern in pediatric dentistry, given their implications for both mental health and long-term oral hygiene behaviors [16]. A large-scale survey in China by Zhao et al. [14], involving over 95,000 students, demonstrated that children who experienced bullying were significantly more likely to suffer from anxiety, depression, and post-traumatic stress symptoms—conditions that visible oral differences could further exacerbate. Moreover, Mao et al. [17] explored how dental anxiety and appearance concerns may influence children’s emotional responses to treatment, suggesting that early negative experiences can erode trust in dental care and compromise future compliance.

While few studies from Saudi Arabia have examined parental satisfaction with SSCs, particularly regarding clinical outcomes [18], there is a notable lack of research on the psychosocial effects of these restorations—especially bullying. The specific link between SSC placement and peer victimization in children remains underexplored in the Saudi context. Given regional cultural norms that emphasize appearance and family reputation, this gap underscores the need for child-centered research that addresses not only clinical success but also the social and emotional impacts of dental treatments.

This study aimed to assess the prevalence of bullying associated with SSCs among Saudi primary school children and to evaluate satisfaction levels among both children and their parents regarding SSC treatment. The first hypothesis proposed that children with SSCs experience higher rates of bullying due to the crowns’ metallic appearance. The second hypothesis proposed that parents would express overall satisfaction with SSC treatment, reflecting their emphasis on functional and clinical outcomes over aesthetic concerns. Understanding these perspectives within the cultural context of Saudi Arabia may guide more child-centered and psychosocially informed dental care.

## 2. Materials and Methods

This school-based cross-sectional observational study was conducted in June 2025 over 4 weeks among children enrolled in private international primary schools in Dammam, Eastern Province, Saudi Arabia. The objective was to evaluate the prevalence of bullying associated with SSCs and to assess both child and parental satisfaction with SSCs as a treatment. The study adhered to the ethical principles of the Declaration of Helsinki and received approval from the Standing Committee of Bioethics Research at Prince Sattam bin Abdulaziz University (Approval No. SCBR-536/2025; 25 May 2025). Written informed consent was obtained from all parents, and assent was collected from the participating children before data collection.

In collaboration with the Ministry of Education, a list of private schools was obtained from which two schools were randomly selected using a simple random sampling technique (lottery draw). Within each selected school, all eligible children aged six to ten years who had received at least one SSC on a primary molar were screened. Inclusion criteria required that the child had received at least one SSC on a primary molar for at least six months and was medically fit. Children were excluded if they had special educational needs, systemic illnesses, psychological or behavioral disorders, or a history of dental trauma.

The required sample size was calculated using G*Power software (version 3.1.9.4), based on an anticipated moderate effect size (Cohen’s d ≈ 0.51), an alpha level of 0.05, and a statistical power of 80%. This calculation yielded a minimum required sample of 120 participants. Of 130 eligible children, 123 agreed to participate, thereby meeting the required sample size.

The parent and child questionnaires used in the study were adapted from previously validated pediatric dentistry instruments, with modifications to assess perceptions of SSCs [8]. Ten pediatric dentistry specialists reviewed the questionnaire’s content and, as a result, questions were reworded for clarity and age-appropriateness. The content validity of the child and parent questionnaires was confirmed using the Content Validity Index (CVI), achieving an acceptable level of 0.9. Reliability testing of the attitude-related items was conducted following pilot testing to assess internal consistency, yielding a Cronbach’s alpha of 0.81. An information pack explaining the study was first distributed to school principals, who then shared it with parents ahead of the survey. The final validated children’s questionnaire consisted of four basic demographics questions (age, sex, school grade, and SSC status), seven binary (yes/no) questions addressing SSC-related bullying (verbal, physical, and social), and perceived psychological and academic impacts over the past six months. The parent questionnaire included three yes/no items evaluating general satisfaction with the SSCs. On the designated survey day, child–parent pairs completed the questionnaires simultaneously at school, with trained dental interns assisting to standardize administration. The surveys were anonymized to minimize response bias and linked parent–child responses using unique study codes.

### Statistical Analysis

Data analysis was conducted using SPSS software (version 21.0; IBM Corp., Armonk, NY, USA). Descriptive statistics summarized demographic characteristics and study variables. Chi-square tests, with Fisher’s exact test applied, when necessary, assessed associations between categorical variables such as gender, bullying experience, and satisfaction levels. A *p*-value of ≤0.05 was considered statistically significant.

## 3. Results

Among 123 children, nearly half (48.8%; n = 60) preferred that others not ask about their SSC, suggesting some degree of self-consciousness. Although a majority reported liking the SSC immediately after placement (59.3%; n = 73), aesthetic endorsement was lower: only 35% (n = 43) liked its shape, and 29.3% (n = 36) liked its color. Peer-related consequences were notable: 29.0% (n = 36) reported being bullied because of the SSC, 8.9% (n = 11) reported school absence, 8.9% (n = 11) perceived discrimination attributable to the crown, and 10.6% (n = 13) believed bullying would harm their school performance. These findings indicate moderate immediate acceptance of SSCs but comparatively limited aesthetic satisfaction and meaningful social impacts for a substantial minority (Table 1).

Table 2 summarizes SSC-related bullying. Overall, 39.0% (48/123) reported being bullied because of their SSC. Rates were similar by sex—40.0% of boys (24/60) versus 38.1% of girls (24/63); this difference was not statistically significant (*p* = 0.829). The majority reported no bullying (61.0%, 75/123). In addition, 23.6% (29/123) reported having a nickname, which was more frequent among boys than girls (*p* < 0.001).

**Figure 1:**
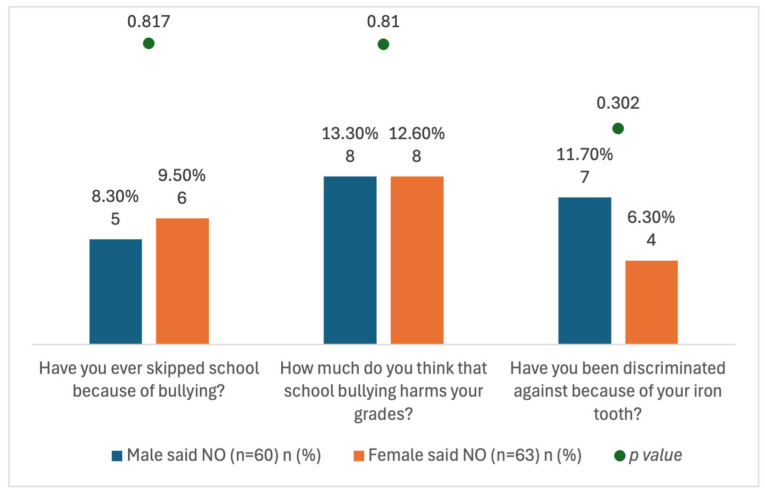
The relation between children’s school performance and gender in bullied children.

Figure 1 summarizes school attendance and academic impact. Overall, attendance was high; only 8.9% (11/123) reported skipping school due to the SSC, with comparable rates among boys (8.3%, 5/60) and girls (9.5%, 6/63). Perceived discrimination at school attributable to the SSC/bullying was reported by 8.9% (11/123) [boys 11.7% (7/60), girls 6.3% (4/63)]. Regarding academic performance among 16 children, 2.4% (3/123) reported that bullying severely harmed their grades, 10.6% (13/123) reported a slight impact, and the majority indicated no academic effect (91.1%, 112/123).

**Figure 2:**
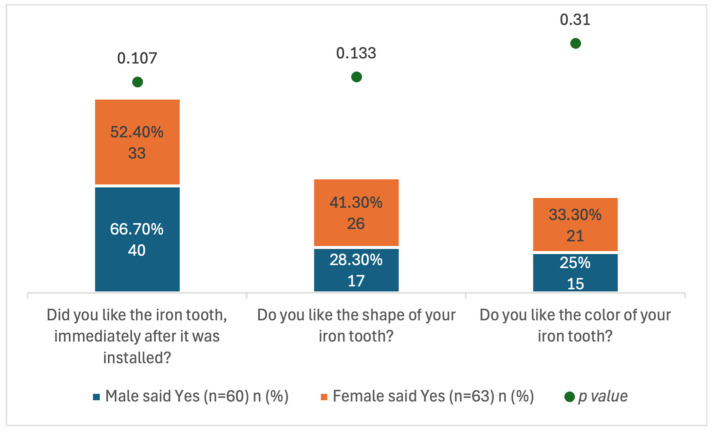
Children’s Aesthetic Satisfaction.

Figure 2 summarizes dentofacial perceptions. Children were asked whether they liked the shape and color of the SSC-treated tooth and its overall appearance. Overall, 59.3% (73/123) reported liking the tooth’s appearance, with no significant difference by sex (*p* > 0.05).

Table 3 summarizes parental perceptions of the stainless-steel crown (SSC). Most parents liked their child’s crown (69.1%, 85/123), and an even larger proportion reported being satisfied with its appearance immediately after placement (72.4%, 89/123). In contrast, fewer parents felt that their child had accepted the crown well (42.3%, 52/123), with 57.7% (71/123) indicating the opposite. Taken together, parents generally viewed the SSC favorably at placement, but perceived lower child acceptance relative to their own liking and immediate post-placement satisfaction.

## 4. Discussion

This study explored the prevalence of bullying associated with SSCs among primary school children in Dammam, Saudi Arabia, while also evaluating satisfaction levels among both children and their parents. The findings supported the hypothesis that the use of SSCs is linked to heightened experiences of bullying and reduced aesthetic satisfaction among children. Full-coronal restorations remain a fundamental component of managing childhood caries, with several options demonstrating varying clinical performance. Modern lifestyle shifts increased social exposure, and pervasive media influence have contributed to children developing aesthetic awareness at a younger age [19]. This heightened concern with appearance—once primarily an adult consideration—has extended into pediatric dentistry, leading to a growing demand for esthetically pleasing alternatives to SSCs, such as composite resins and zirconia crowns.

Verbal victimization has been highlighted by prior research as the subtype most consistently linked to subsequent impacts on well-being and domains relevant to oral-health–related quality of life. Verbal aggression is easy to perpetrate, can be repeated frequently, and often carries low immediate cost to the perpetrator; victims may therefore experience chronic stigma and erosion of belonging consistent with social identity theory, leading to emotional distress and lower self-esteem, which in turn are associated with less optimal oral-health behaviors and day-to-day functioning [20]. Our study did not administer a validated OHRQoL scale, so we reference these pathways from the literature rather than claim them from our dataset. Nevertheless, the pattern of child-reported aesthetic reservations and social exposure is compatible with these mechanisms.

Bullying remains a global concern, with international studies reporting prevalence rates ranging from 8.6% to 45.2% among boys and 4.8% to 35.8% among girls. In Mexico, 52.7% of elementary and 28% of university students reported being bullied. In the Kingdom of Saudi Arabia (KSA), the first nationally representative study found bullying and physical violence rates of 24% and 21%, respectively [21]. In comparison, our study revealed a slightly higher prevalence of 39%. This aligns with prior findings that peer victimization is inversely related to social support from family and peers. At the same time, poor coping skills and lack of independence can increase vulnerability to bullying. Among the 123 participants, 29% of children reported experiencing bullying related to their SSCs. This relatively high prevalence is comparable to rates reported in other Middle Eastern countries, such as Oman (38.9%) and Lebanon (33.6%). In contrast, studies from high-income regions have reported significantly lower bullying prevalence associated with dental appearance, including the United Arab Emirates (20.9%), the United Kingdom, and the United States, where rates typically range between 8 and 15% [22,23]. These discrepancies may reflect cultural differences in how visible dental restorations are perceived.

Our study expands upon earlier work by exploring both the general acceptance of SSCs and their psychosocial implications, specifically bullying. Utami et al. [24] investigated children’s and parents’ acceptance of SSCs in Indonesia, reporting that 95% of children liked the look of their ‘iron teeth’ and 92.5% of parents believed their child accepted the crown well. Additionally, 70% of the children indicated that they did not mind being asked about their crowns. In contrast, our findings revealed that nearly half of the children (48.8%) felt uncomfortable when questioned about their SSC, and 29% reported being bullied due to their crown. These discrepancies underscore the importance of including psychosocial dimensions, such as peer perceptions and emotional responses, when evaluating restorative outcomes in pediatric dentistry.

Although most children reported regular school attendance, a notable subset experienced educational and psychosocial disruptions: 8.9% reported skipping school due to bullying, and an equal proportion perceived discrimination linked to their SSC. Academic performance was also affected, with 10.6% indicating a slight impact on their grades and 2.4% reporting severe consequences. These findings are consistent with broader research showing that children with oral diseases may miss school due to pain or dental appointments, and may also suffer from discomfort, humiliation, delayed cognitive development, low self-esteem, and challenges in daily functioning [25]. These outcomes highlight that, while many children adapt, a significant minority experience distress and social burden.

Zimmerman et al. [26] identified four key factors influencing parental attitudes toward pediatric restorative materials: aesthetics, cost, toxicity concerns, and durability. In line with these findings, our study also found that aesthetic concerns were a central factor shaping parental satisfaction with SSCs. While Zimmerman et al. reported that families from higher socioeconomic backgrounds tend to prioritize esthetics more heavily, our study did not assess socioeconomic status, limiting our ability to examine such associations. Nevertheless, these insights underscore the broader need to consider parental perspectives when planning pediatric restorative care, particularly for visible restorations such as SSCs. Furthermore, the role of shared decision-making was highlighted, as earlier studies have shown that approximately 43% of dentists consider parental preferences before treatment planning, underscoring the importance of involving parents in restorative decisions to align care with family expectations.

A recent study by Jain et al. [19] found that parental satisfaction with zirconia crowns was significantly higher, reaching 96% (*p* = 0.03), largely due to their superior aesthetics. In contrast, parental satisfaction with SSCs in our study was notably lower, with only 72.4% of parents satisfied with the crown’s appearance. This disparity highlights the influence of esthetic expectations on treatment acceptance and underscores the importance of aligning restorative choices with both clinical indications and parental preferences.

A recent study comparing satisfaction with SSCs and composite resin restorations reported that children’s satisfaction with restoration color was initially 75% in the SSC group and 85% in the composite resin group. Although this difference was not statistically significant at the time of treatment (*p* = 0.246), a considerable divergence emerged after one year: satisfaction declined to 69% in the SSC group and increased to 90.6% in the composite group (*p* < 0.001). The study also highlighted that while parents’ concerns about color diminished over time, children became more sensitive to aesthetic differences—likely due to peer feedback and increased self-awareness [27]. In our study, 29.3% of children reported satisfaction with the crown color, indicating lower immediate esthetic acceptance than previously reported. In contrast, 69.1% of parents were satisfied with the SSC’s appearance, suggesting a clear perceptual gap between children and caregivers. Unlike the comparative study, our investigation did not include long-term follow-up, which limits insight into whether children’s attitudes toward the crowns improved or worsened over time. Nevertheless, the current findings emphasize the need to consider the child’s evolving psychosocial response to visible restorations beyond the point of placement.

These findings highlight the importance of child-centered clinical decision-making. Dentists should discuss aesthetic alternatives, such as zirconia crowns, especially for visible teeth, and involve both parents and children in treatment planning. A survey of pediatric dentists revealed that 87% of parents are concerned about the esthetics of even posterior restorations, reflecting rising expectations for visually acceptable outcomes [28]. Additionally, school-based anti-bullying initiatives and teacher training are essential to reduce appearance-related teasing and support affected children.

Despite its contributions, this study has limitations. The cross-sectional design precludes causal inferences, and the sample was restricted to two private schools in one city, limiting generalizability. The reliance on self-report data and binary response options may have limited sensitivity in capturing nuanced psychosocial effects. The lack of clinical dental examinations and the English-only questionnaire may have introduced selection or reporting bias. Clinical examinations were considered but were not feasible on-site and might have inhibited disclosure; therefore, brief English self-report instruments were used to maximize participation and convenience.

Despite these constraints, this study offers valuable insight into the psychosocial impact of SSCs in a culturally specific context. It highlights the gap between parental perceptions and child experiences and reinforces the importance of child-centered care in pediatric dentistry. Future research should explore the long-term psychological effects of visible dental restorations and compare outcomes across different restorative materials. Longitudinal and qualitative approaches may further elucidate how children process and adapt to appearance-related challenges over time.

This study examined the prevalence of bullying associated with SSCs among primary school children in Dammam, Saudi Arabia, and evaluated child and parental satisfaction. Findings supported both hypotheses: SSCs were linked to bullying—primarily verbal (58.1%)—and children reported lower aesthetic satisfaction compared to parents. While 59.3% of children initially liked their crowns, only 35.0% approved the shape and 29.3% the color, with nearly half feeling self-conscious when asked about them. In contrast, parents reported higher satisfaction (69.1%) and appearance approval (72.4%), though only 42.3% believed their child had truly accepted the crown—revealing a gap between parental priorities and children’s psychosocial experiences.

## 5. Conclusions

This study demonstrates that SSCs, while functionally effective and widely accepted by parents, are associated with significant psychosocial concerns among children, including bullying and aesthetic dissatisfaction. These findings highlight the importance of considering the child’s emotional and social experiences in treatment planning. Children wearing SSCs may be at increased risk of peer-related stigma, particularly in school environments where appearance plays a critical role in social integration. Pediatric dentists should therefore prioritize shared decision-making, actively include the child’s perspective, and offer aesthetic restorative alternatives—such as zirconia crowns—when appropriate. Enhancing parental awareness, improving school-based education about dental treatments, and fostering communication between dental professionals and families can help reduce the psychosocial burden and support the child’s overall well-being.

## Figures and Tables

**Table 1:** Children’s responses to the questionnaire.

Children Questions	Yes (n (%))	No (n (%))
1. Do you mind people asking about your iron tooth?	60 (48.8%)	63 (51.2%)
2. Did you like the iron tooth immediately after it was installed?	73 (59.3%)	50 (40.7%)
3. Do you like the shape of your metallic tooth?	43 (35%)	80 (65%)
4. Do you like the color of your metallic tooth?	36 (29.3%)	87 (70.7%)
5. Have you been bullied by any student(s) from school in the past month because of your iron tooth?	48 (39%)	75 (61%)
6. Have you ever skipped school because of bullying?	11 (8.9%)	112 (91.1%)
7. Have you been called names for your iron tooth?	29 (23.6%)	94 (76.4%)
8. How much do you think that school bullying harms your grades?	Severely affected 3 (2.4%) Slightly affected 13 (10.6%)	Not affected 107 (87%)
9. Have you been discriminated against because of your iron tooth?	11 (8.9%)	112 (91.1%)

**Table 2:** Number of students who were victims of bullying or reported bullying other students.

	Males (n = 60) n (%)	Females (n = 63) n (%)	*p* Value	Total (n = 123) n (%)
Have you been bullied?	24 (40%)	24 (38.1%)	0.829	48 (39%)

**Table 3:** Parents’ questions.

Parents Questions	Yes (n (%))	No (n (%))
1. Do you like your child’s metallic crown?	85 (69.1%)	38 (30.9%)
2. Has your child accepted his/her metallic crown well?	52 (42.3%)	71 (57.7%)
3. Were you satisfied with the appearance of the metallic crown, immediately after it was installed?	89 (72.4%)	34 (27.6%)
Total	123	

## Data Availability

The original contributions presented in this study are included in the article. Further inquiries can be directed to the corresponding author.
